# Vaginal and Anal Microbiome during *Chlamydia trachomatis* Infections

**DOI:** 10.3390/pathogens10101347

**Published:** 2021-10-19

**Authors:** Stefano Raimondi, Francesco Candeliere, Alberto Amaretti, Claudio Foschi, Sara Morselli, Valeria Gaspari, Maddalena Rossi, Antonella Marangoni

**Affiliations:** 1 Department of Life Sciences, University of Modena and Reggio Emilia, 41121 Modena, Italy; stefano.raimondi@unimore.it (S.R.); francesco.candeliere@unimore.it (F.C.); alberto.amaretti@unimore.it (A.A.); maddalena.rossi@unimore.it (M.R.); 2 Microbiology, Department of Experimental, Diagnostic and Specialty Medicine (DIMES), University of Bologna, 40138 Bologna, Italy; sara.morselli6@unibo.it (S.M.); antonella.marangoni@unibo.it (A.M.); 3 Dermatology Unit, Istituto di Ricovero e Cura a Carattere Scientifico (IRCCS), St. Orsola Malpighi University Hospital, 40138 Bologna, Italy; valeria.gaspari@aosp.bo.it

**Keywords:** *Chlamydia trachomatis*, microbiome, vagina, anus, STIs, women’s health

## Abstract

**Background.***Chlamydia trachomatis* (CT) is the agent of the most common bacterial sexually transmitted infection worldwide, with a significant impact on women’s health. Despite the increasing number of studies about the vaginal microbiome in women with CT infections, information about the composition of the anal microbiome is still lacking. Here, we assessed the bacterial community profiles of vaginal and anal ecosystems associated or not with CT infection in a cohort of Caucasian young women. **Methods.** A total of 26 women, including 10 with a contemporary vaginal and ano-rectal CT infection, were enrolled. Composition of vaginal and anal microbiome was studied by 16S rRNA gene profiling. Co-occurrence networks of bacterial communities and metagenome metabolic functions were determined. **Results.** In case of CT infection, both vaginal and anal environments were characterized by a degree of dysbiosis. Indeed, the vaginal microbiome of CT-positive women were depleted in lactobacilli, with a significant increase in dysbiosis-associated bacteria (e.g., *Sneathia, Parvimonas, Megasphaera*), whereas the anal microbiota of CT-infected women was characterized by higher levels of *Parvimonas* and *Pseudomonas* and lower levels of *Escherichia*. Interestingly, the microbiome of anus and vagina had numerous bacterial taxa in common, reflecting a significant microbial ‘sharing’ between the two sites. In the vaginal environment, CT positively correlated with *Ezakiella* spp. while *Gardnerella vaginalis* co-occurred with several dysbiosis-related microbes, regardless of CT vaginal infection. The vaginal microbiome of CT-positive females exhibited a higher involvement of chorismate and aromatic amino acid biosynthesis, as well as an increase in mixed acid fermentation. **Conclusions.** These data could be useful to set up new diagnostic/prognostic tools, offering new perspectives for the control of chlamydial infections.

## 1. Introduction

*Chlamydia trachomatis* (CT) is the agent of the most common bacterial sexually transmitted infection (STI) worldwide, with a significant clinic, economic, and public health impact [1]. Urogenital CT infections in women (i.e., urethritis and cervicitis) are often asymptomatic and, when untreated, can lead to several sequelae and complications, including pelvic inflammatory disease, tubal infertility, and ectopic pregnancy [2]. Moreover, urogenital infections are associated with an increased likelihood of HIV infection transmission and acquisition [3].

Besides the common urogenital localizations, *Chlamydia* can be found at extra-genital sites, such as pharyngeal and ano-rectal mucosa, on the basis of the sexual repertoires of the couples [4,5]. The prevalence of anal CT infections can be up to 5–15% in female populations that generally report unsafe intercourse and/or multiple sexual partners [6]. These infections are often asymptomatic or characterized by non-specific symptoms, acting as an important reservoir for further transmission [7,8].

The correlation between CT infection and composition of the cervico-vaginal microbiome has recently gained particular attention due to CT prevalence and clinical impact [9,10,11]. Some cross-sectional studies have demonstrated that bacterial vaginosis (BV) characterized by a dysbiosis status, with depletion of lactobacilli and increase in other anaerobic species, is an independent risk factor for STI acquisition, including genital CT infections [12,13]. Indeed, the vaginal environment of CT-positive women is often characterized by a decrease in *Lactobacillus* spp., together with a significant increase in BV-associated bacterial taxa, such as *Megasphaera* spp., *Atopobium vaginae*, *Gardnerella vaginalis*, *Dialister* spp., and *Prevotella* spp. [14,15,16]. Nonetheless, many aspects of the vaginal microbiome during genital CT infection remain to be fully elucidated, and to the best of our knowledge, no information about the composition of the anal microbiome during ongoing CT infections in women is currently available.

To fill this gap, we compared vaginal and anal microbiome composition in a cohort of 10 young women with contemporary vaginal/rectal CT infection and 16 CT-negative women. These data could be crucial to better understand the pathogenesis of genital and extra-genital CT infections, offering new perspectives for their prophylaxis and treatment.

## 2. Results

### 2.1. Study Population and Samples Analyzed

A total of 26 women were enrolled in the study: 10 patients (38.5%) showed a contemporary genital and ano-rectal CT infection (i.e., CT-positive group), while the other 16 (61.5%) were negative for CT at both anatomical sites (i.e., CT-negative group). Thus, 10 coupled CT-positive vaginal (VS+) and anal samples (AS+), as well as 16 coupled CT-negative vaginal (VS−) and anal samples (AS−) were analyzed. No significant differences were found in the mean age between the two groups (CT-positive: 22.8 ± 1.7, CT-negative: 22.3 ± 2.8; *p* = 0.6).

### 2.2. Diversity of Vaginal and Anal Microbiome

The 16S rRNA gene profile of vaginal (VS, n = 26) and anal (AS, n = 26) samples encompassed a total of 1,028,693 quality-trimmed sequences, from 6412 to 54,485 reads per sample. The sequences were dereplicated into 1287 amplicon sequence variants (ASVs) matching a reference sequence in the Silva database and collapsed at the 7th level of taxonomic annotation (i.e., the species, if available) into 284 operational taxonomic units (OTUs).

Richness and evenness, described by Chao1, Shannon, and Pielou indices (Figure S1), were higher in anal samples than in vaginal ones (*p* < 0.0001). The richness (Chao1 index) of both anal and vaginal microbiome was similar in CT-positive samples compared to CT-negative ones (*p* > 0.05), while the evenness (Pielou index) was higher in VS+ than in VS− (*p* < 0.01).

**Figure 1:**
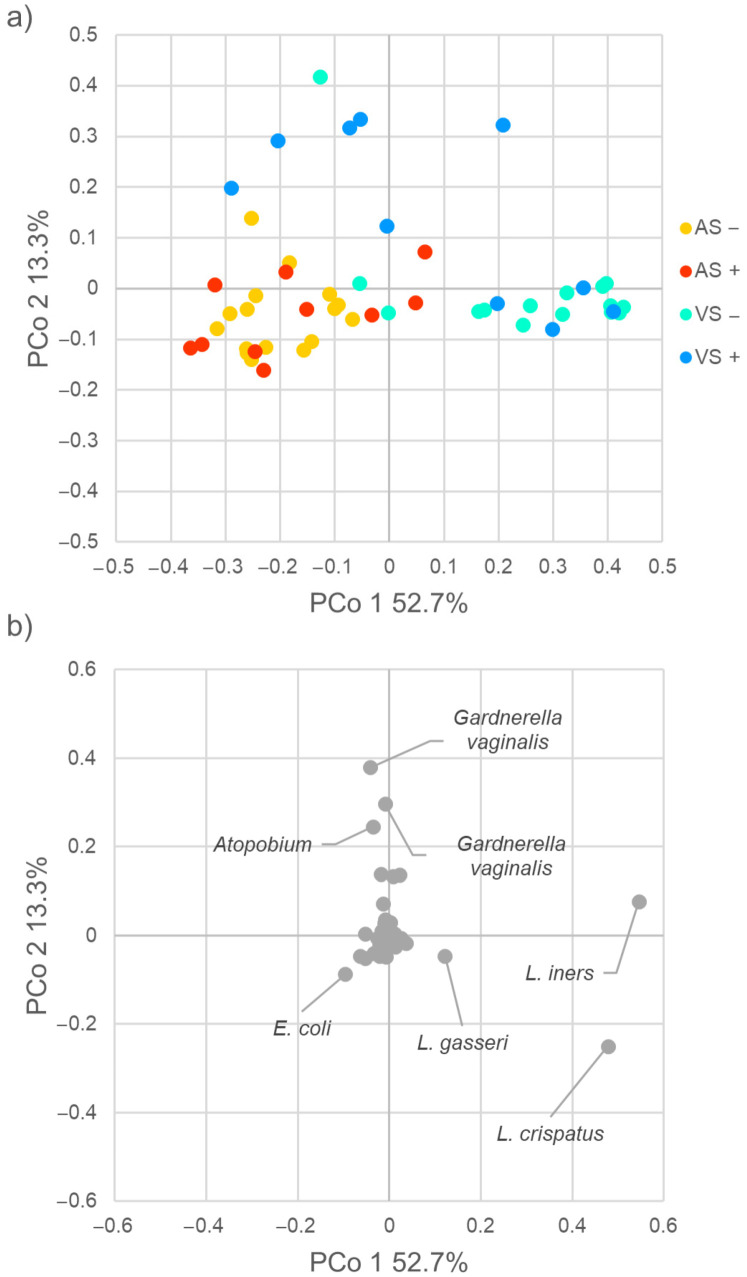
β-diversity of the vaginal and anal microbiome. The microbial composition of vaginal (VS) and anal samples (AS) of CT-positive (+) and negative (−) women was analyzed. (**a**) Principal coordinates analysis (PCoA) plot based on weighted Unifrac distance. Each point corresponds to a sample. (**b**) PCo1–PCo2 visualization of the contribution of each ASV. ASVs exerting the highest effect on PCo1 and/or PCo2 are labelled.

The beta-diversity among samples was assessed by weighted UniFrac metrics. In the plot of principal coordinate analysis (PCoA) reporting the two most informative dimensions (principal coordinate 1, PCo1; principal coordinate 2, PCo2), anal and vaginal samples clustered separately (PERMANOVA, *p* = 0.001), with anal samples grouping together at low values of PCo1 and PCo2 (*p*= 0.744, Figure 1a). Vaginal swabs were split into two groups (*p* = 0.023), with VS+ mostly defined by high PCo2 values and VS− by high PCo1 ones. The ASVs that mainly contributed to positive PCo2, associated with VS+ metagenomes, belonged to *Atopobium* and *Gardnerella vaginalis*, while lactobacilli ascribed to the species *Lactobacillus crispatus*, *Lactobacillus iners*, and *Lactobacillus gasseri* were the main drivers of positive PCo1, associated with VS− samples (Figure 1b). Sequences assigned to the species *Escherichia coli* were frequent in AS samples and negatively contributed to both PCo1 and PCo2.

### 2.3. Taxonomic Composition of Vaginal and Anal Bacterial Communities

**Figure 2:**
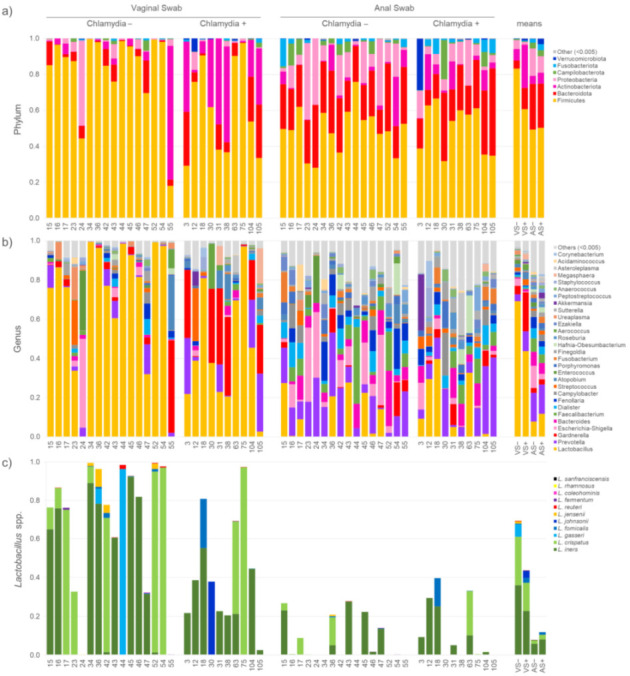
Taxonomic composition of the vaginal and anal microbiome. Stacked bar charts representation of microbiota composition (relative abundance) at phylum (**a**), genus (**b**), and *Lactobacillus* spp. (**c**) level. On the left, each vaginal and anal sample, stratified for CT positivity, is represented individually, whereas on the right the mean relative abundances for each group is depicted. Taxa present at relative abundances <0.5% are grouped in the “Other” category.

In vaginal samples, Firmicutes was the predominant phylum (60.8% and 83.2% in VS+ and VS−, respectively), followed by Actinobacteriota (23.8% and 6.4%), Bacteroidota (11.8% and 4.6%), and Proteobacteria (1.7% and 4.8%). Firmicutes were the most abundant also in anal samples (50.3% and 49.4% in AS+ and AS−, respectively), followed by Bacteroidota (24.7% and 25.4%), Proteobacteria (9.2% and 15.3%), and Actinobacteriota (6.0% and 4.4%) (Figure 2a).

*Lactobacillus* was the sole genus shared by all the VS samples, ranging from 0.1 to 99.5% (Figure 2b), with a mean relative abundance of 43.5% in VS+ and 69.3 in VS−. In several samples, *Lactobacillus* overwhelmed the other genera by more than one magnitude unit, with the sole exception of Gardnerella that accounted, on average, for 10.2% of the bacterial abundance. *Prevotella* was another abundant and recurrent taxon, occurring in 24 vaginal samples with a mean abundance of 6.1%. *Escherichia-Shigella*, *Atopobium*, and *Streptococcus* exceeded the 2% of abundance but were more sporadically detected (9 out of 26 vaginal samples). *Finegoldia*, *Peptoniphilus*, *Anaerococcus*, and *Candidatus Zinderia*, although frequently detected (20 to 22 out of 26 samples), represented a minor component of the vaginal microbiota (<0.1%). *Lactobacillus* was scarce in two vaginal swabs (0.1 and 2.5% in VS−55 and VS+105, respectively), both dominated by *Gardnerella vaginalis* (46.6% and 25.6% in VS−55 and VS+105, respectively). *Lactobacillus* was especially low also in VS−24 (0.1%), where *E. coli* and *Enterococcus* faecalis outnumbered other taxa (40.1% and 30.0%, respectively).

A total of 32 ASVs ascribed to “Lactobacillus_uncultured” were further assigned by single BLAST alignment to the species *L. crispatus, L. gasseri, L. fornicalis, L. coleohominis, L. acidophilus, L. iners, L. johnsonii*, and *L. reuteri*, and the corresponding relative abundance was determined (Figure 2c). *L. crispatus* and *L. iners* were the most common and abundant species of lactobacilli in vaginal microbiota. *L. crispatus* was identified in 20 out of 26 samples and represented 14.5% in VS+ and 24.9% in VS− of total bacteria. *L. iners*, detected in 14 VS, accounted for 22.7% in VS+ and 36.1% in VS−. *L. reuteri* and *L. fornicalis*, were observed in eight VS but represented a minor bacterial component: the former was <0.2% in both groups and the latter was 2.6% in VS+ and 0.4% in VS−. *L. gasseri* was an occasional species detected only in four VS− samples, outnumbering all other bacteria in VS− 44 (96.4%). None of the identified species of Lactobacillus showed significantly different abundance (*p* > 0.05) between CT-positive and negative vaginal samples.

According to VALENCIA algorithm, the bacterial communities of the 26 VS were classified in seven community state types (CSTs), type III-A and III-B (five and six samples, respectively) being the most represented ones (Figure S2). These two CSTs were dominated by the genus *Lactobacillus* and, in particular, by *L. gasseri*. CST type IV-B encompassed six samples, generally CT-positive (five out six) and was characterized by a low relative abundance of lactobacilli. On the other side, in all the vaginal swabs ascribed to CST III-A, CT was never detected.

The anal bacterial community was distributed among 205 genera, 23 of which at mean abundances greater than 1%. *Prevotella* was the dominant genus (14.7%), followed by Lactobacillus, accounting for 9.3% of total bacteria. *Prevotella, Bacteroides*, and *Peptoniphilus* were observed in all the AS. *Dialister, Streptococcus, Escherichia-Shigella, Campylobacter,* and *Anaerococcus* were absent in 1 or 2 samples out of 26.

Among the 284 different OTUs identified within the 52 anal and vaginal samples, 193 (68%) were shared by the two sites (Figure S3). Among them, 127 OTUs were detected in vaginal and anal samples of the same subjects. Most of these OTUs were components of the female urogenital microbiota, such as *Lactobacillus, Gardnerella, Streptococcus, Staphylococcus, Corynebacterium*, and *E. coli* [17]. It is noteworthy that no significant differences in the number of OTUs shared in VS and AS were found based on the presence or absence of CT.

**Figure 3:**
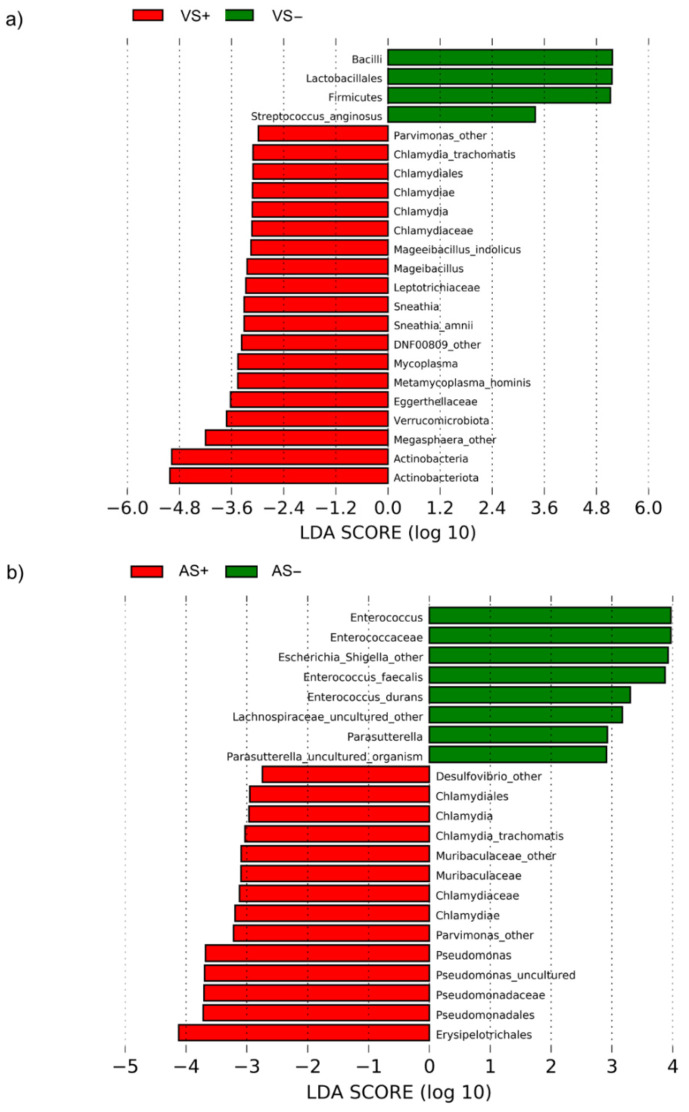
Distinctive taxonomic features characterizing CT-positive and negative samples. LDA score provided by LEfSe algorithm revealed the members of the microbial communities that were different between CT-positive and CT-negative groups, for vaginal (**a**) and anal (**b**) samples.

LEfSe algorithm was applied to reveal distinct features of the metagenomes characterized by the presence or absence of CT in vaginal samples. Overall, 23 taxa exhibited significantly different abundances (*p* < 0.05) between VS− and VS+ (Figure 3a and Figure S4). Four taxa were overabundant in VS−, including the order Lactobacillales and the genus *Streptococcus anginosus*. On the other side, 14 taxa characterized VS+ samples, including the genera *Megasphera* and *Parvimonas* and the species *Metamycoplasma hominis*, *Sneathia amnii*, and *Mageibacillus indolicus*.

In anal metagenomes (Figure 3b and Figure S4), *Enterococcus* (*E. faecalis* and *E. durans*), *Escherichia-Shigella*, and *Parasutterella* were more abundant in AS−, while *Desulfovibrio*, *Parvimonas*, and *Pseudomonas* were more abundant in AS+.

### 2.4. Taxonomic Co-Abundance Clusters

**Figure 4:**
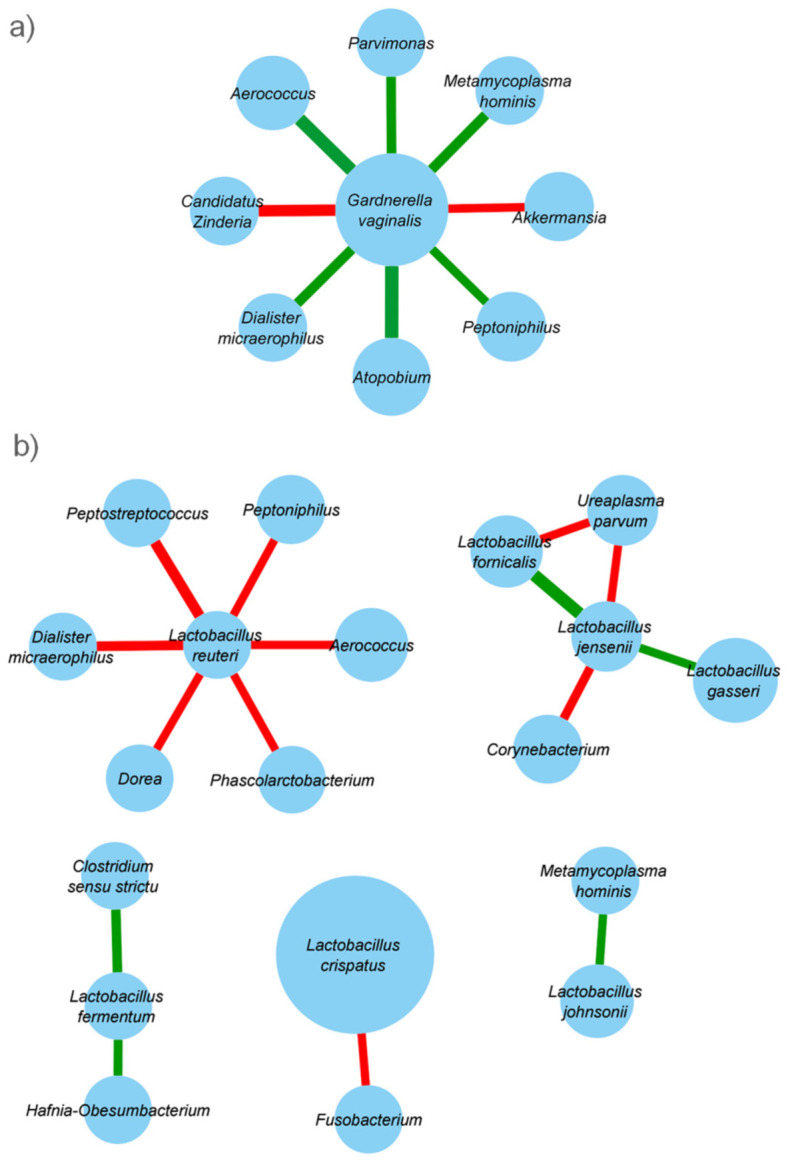
Taxonomic co-abundance clusters. Co-occurrence networks of bacterial communities of vaginal samples, irrespective of *Chlamydia* positivity. Significant correlations between *G. vaginalis* (**a**), *Lactobacillus* spp. (**b**) and the other bacterial taxa identified in vaginal samples were calculated using SCNIC and visualized with Cytoscape. Green and red bars represent positive and negative correlations, respectively. The thickness of the bar is proportional to the correlation coefficient *r*.

To evaluate interspecies interactions, co-occurrence networks of bacterial communities were reconstructed in vaginal samples, irrespective of CT positivity. Correlations between CT*, G. vaginalis, Lactobacillus* spp., and the other bacterial taxa identified in vaginal samples were calculated using SCNIC and visualized with Cytoscape (Figure 4a).

CT co-occurred with *Ezakiella*. *G. vaginalis* network exhibited predominantly positive correlations, indicating cooperative relationships with vaginal bacterial taxa such as *Parvimonas, Anaerococcus, Metamycoplasma hominis, Dialister, Atopobium*, and *Peptoniphilus*. On the other hand, lactobacilli mainly showed negative correlations with other bacterial taxa (Figure 4b). For instance, *Lactobacillus reuteri* was negatively related to the presence of several BV-associated bacteria (e.g., *Peptostreptoccocus, Peptoniphilus, Dialister, Anaerococcus*), and *L. crispatus* to *Fusobacterium*. Some *Lactobacillus* species (i.e., *L. gasseri. L. fornicalis, L. jensenii*) were positively related to each other.

### 2.5. Predicted Metabolic Functions

**Figure 5:**
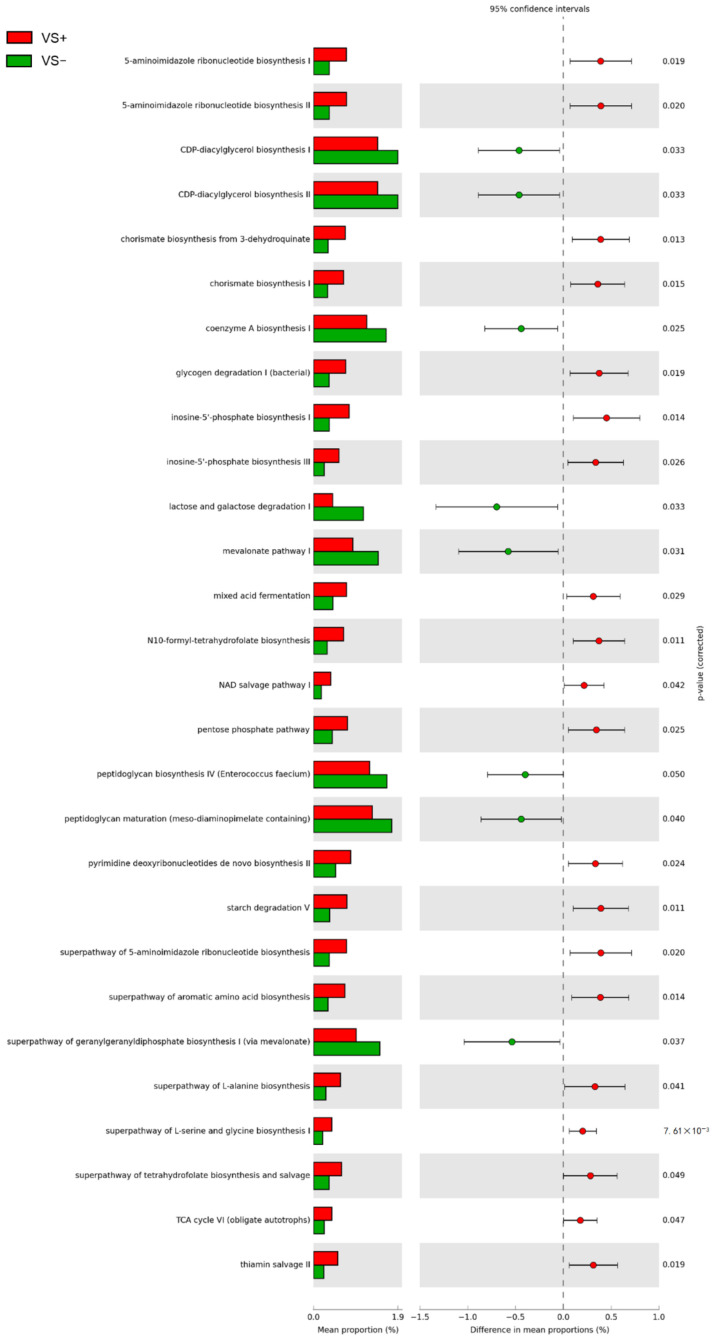
Metabolic functional analysis prediction. The metabolic pathways reconstructed from 16S rRNA gene profile of vaginal samples using PICRUSt2 and presenting significant (*p* < 0.05) differential abundance between CT-positive and negative samples were reported.

Metagenome reconstruction by 16S rRNA gene profiling of vaginal samples was exploited to predict the metabolic functions with PICRUSt2 and MetaCyc database. A total of 371 pathways were identified, 36 of which showed significantly different abundance (*p* < 0.05) between VS+ and VS− groups (Figure 5).

The predicted pathways characterizing VS− were involved in central catabolism of lactose and galactose. Furthermore, modules of the biosynthetic pathways for peptidoglycan, geranyl-geranyl-diphosphate and mevalonate, coenzyme A, and CDP-diacylglycerol were more represented in VS− samples. On the other side, the pentose phosphate shunt, the mixed acid fermentation, portions of the TCA cycle, glycogen, and starch degradation were enriched in VS+ samples. The pathways involved in amino acid biosynthesis also characterized VS+ metabolism, in particular the chorismate and super-pathway for the biosynthesis of aromatic amino acids, glycine, serine, and alanine.

Among the 363 pathways predicted by PICRUSt2 in anal samples, only 10 presented significant differences between AS+ and AS− groups (*p* < 0.05), all being more abundant in AS− samples (Figure S5). Differential abundance was observed mainly for degradation of aromatic compounds and amino acids (catechols and phenylpropanoids) and for pathways related to enterobacteria (sulfoglycolysis and enterobactin biosynthesis).

## 3. Discussion

Composition of vaginal and anal microbiome in a cohort of sexually active young women was investigated comparing the bacterial composition of CT-infected women (n = 10) to a negative control group (n = 16).

Both vaginal and anal ecosystems were characterized by a degree of dysbiosis in case of CT infection, with several changes in the microbial composition compared to CT-negative women. These modifications may occur as a result of CT infection or be a predisposing factor, favoring chlamydial survival and replication.

In line with previous findings [18], the vaginal microbiome of CT-negative women was mainly constituted by Firmicutes, dominated at a lower taxonomic level by members of genus *Lactobacillus*. It is worth mentioning that, in some CT-negative subjects, even though asymptomatic, lactobacilli were diminished and replaced by BV-associated bacteria (e.g., *Gardnerella*) or intestinal-derived microorganisms (e.g., *Escherichia*, *Enterococcus*). Furthermore, several conditions of vaginal dysbiosis, such as BV or aerobic vaginitis, can be completely asymptomatic, even in presence of significant alterations of the microbial homeostasis [19,20]. As formerly reported [11,12], *Lactobacillus* showed a significant decrease in CT-positive women, with a contemporary increase in *Sneathia, Parvimonas, Metamycoplasma*, and *Megasphaera*. All these microorganisms have been associated with a BV status, strengthening the idea that the vaginal environment during chlamydial infections is often characterized by a dysbiotic condition [9]. The literature suggests that women with genital CT infection are most likely to be in CST IV, characterized by a reduction in *Lactobacillus* species [21]. Consistently, in our study cohort, a larger proportion of CT-positive women, as compared with the uninfected group, had a microbial profile similar to that of CST IV.

When focusing on *Lactobacillus* genus, we found a high predominance of *L. crispatus* and *L. iners* species in both VS− and VS+ groups. *L. crispatus* has been recognized as a hallmark of vaginal health and eubiosis, while *L. iners* is considered to be a ‘transitional’ species, colonizing after perturbations of the vaginal environment [22]. Even though higher levels of *L. iners* have been previously associated with CT infection [12], in our cohort, this species did not show significantly different abundance between CT-positive and negative vaginal samples. Nevertheless, it has been recently shown that *L. iners* is the dominating taxon in a large subset of women worldwide, being its presence associated with unprotected sex practices and a higher number of sexual partners [23]. Moreover, among reproductive age women, younger women are more likely to have *L. iners*-dominated vaginal communities than older women [24].

Anal samples were mainly characterized by the presence of Firmicutes, Proteobacteria and Bacteroidota, being *Prevotella* one of the most represented genus. This taxonomic composition is similar to previously described bacterial communities of the ano-rectal mucosa in subjects reporting unsafe anal intercourse [25,26]. Recent studies revealed that the microbiota in stools and anal swabs was similar and that differences between swab and stool samples within the same subject were significantly lower than those from samples between subjects [27,28]. Nevertheless, divergences in the relative abundance of taxa were reported, such as overrepresentation of Enterobacteriaceae and Bacillaceae in swabs and Clostridiales in stools. In accordance with these findings, AS metagenome showed a shift in composition compared to stool microbiota of a similar cohort of subjects, equivalent for age and geographical origin [29], revealing an increased amount of Proteobacteria and Actinobacteriota (mainly *Gardnerella* and *Escherichia-Shigella*, respectively). The enrichment in members of the Prevotellaceae family has been previously associated with mechanical microtrauma and deposition of semen resulting in transient damages and inflammatory responses able to affect the commensal microbiota [25].

Interestingly, both in CT-positive and negative women, the microbiome of anus and vagina had numerous bacterial taxa (more than 60%) in common, reflecting a significant microbial ‘sharing’ between the two sites. It is well known that the ano-rectal region is a significant reservoir of ‘health-promoting’ bacteria, *Lactobacillus* species in the rectum being possible contributors to the maintenance of a ‘normal’ vaginal microflora [30]. On the other hand, the ano-rectal region can be a source of potentially harmful microbes (e.g., *Candida* spp., Enterobacterales), able to easily reach and infect the vaginal mucosa [31]. The significant ‘microbial translocation’ between the anal and vaginal sites is also pointed out by the important contribution of non-sexual inoculation of the ano-rectum with infected cervico-vaginal secretions in case of chlamydial infections [32]. Further studies, including culture-based techniques, will be needed to understand the exact magnitude of microbial translocation between anus and vagina both in presence and in absence of CT and to assess if these bacterial sharing can impact on CT infection and pathogenesis.

Other interesting data emerged from the analysis of anal microbiome composition, stratified for CT positivity. Patients with anal CT infection were characterized by significantly higher levels of *Parvimonas* and *Pseudomonas*, whereas CT-negative women exhibited higher abundances of *Escherichia* and *Enterococcus.* In a previous paper focused on ‘men having sex with men’, we found similar microbial shifts in ano-rectal bacterial composition during sexually transmitted infections due to CT or *N. gonorrhoeae*. Indeed, infected patients were characterized by a depletion of *Escherichia* species, associated with an increase in anaerobic genera, including *Peptoniphilus, Peptostreptococcus*, and *Parvimonas* [26]. Even though the reasons behind these microbial changes are still unknown, we can make several hypotheses: (i) *Chlamydia* could elicit an inflammatory response (i.e., recruitment of natural killer cells and neutrophils with the production of cytokines and metalloproteinases) [33], able to alter the equilibrium of the bacterial communities of the anal microbiota, with the depletion of or increase in some species; (ii) the oxygen consumption by CT-infected cells or by the recruited leukocytes could favor the proliferation of strict anaerobes; (iii) *Chlamydia* can modify specific metabolic pathways for its nutritional requirement, leading to the preferential proliferation of selected microbial species in the anal microbiota.

The depletion of *Escherichia* in the anal microbiota could precede the onset of CT infection, acting as a risk factor for pathogen replication on the ano-rectal site. If so, it would be possible to consider the administration of probiotic *Escherichia* strains as a new antibiotic-sparing approach to prevent chlamydial rectal infections. In this context, probiotic formulations of *E. coli* (e.g., *Escherichia coli* Nissle 1917) have been successfully employed for fighting several infectious agents, such as Salmonella, pathogenic *E. coli, Shigella, Yersinia, Listeria*, and *Candida* [34].

To identify patterns of co-expression in the vaginal microbiota, we established co-abundant genera associations on the whole dataset. Irrespective of CT positivity, *Lactobacillus* was negatively correlated with several BV-associated genera (e.g., *Peptostreptococcus, Peptoniphilus, Dialister*), thus underlining their protective action against the overgrowth of dysbiosis-related microorganisms.

On the other hand, *Gardnerella* occurred together with several dysbiosis-related taxa (e.g., *Peptoniphilus, Dialister, Atopobium, Parvimonas,* and *Metamycoplasma hominis*). These bacterial associations reflect the ability of bacteria to form highly structured polymicrobial biofilms on the vaginal epithelium during BV. Virulent strains of *Gardnerella* spp. initiate the formation of the biofilm and become a scaffolding, to which other BV-associated anaerobes, such as *Atopobium vaginae*, can thereafter attach [35]. Interestingly, **CT** was positively associated with *Ezakiella* spp., a recently described anaerobic Gram-positive species that can be found in human vagina [36].

Significant differences in several functional pathways were found between CT-positive patients and the control group. Among all, a higher involvement of chorismate and aromatic amino acid biosynthesis, as well as an increase in mixed acid fermentation, were predicted at the vaginal level of CT-positive patients. Although non-predictive further studies are needed for a thorough comprehension of the dynamics taking place in the vaginal ecosystem, we can speculate that these variations reflect both the metabolic activities of dysbiosis-associated microbes found during chlamydial infections and the activation of peculiar metabolic pathways employed by *Chlamydia* for its nutritional requirements.

Intracellular growth and pathogenesis of *Chlamydia* species is controlled by the availability of the aromatic amino acid tryptophan [37]. In particular, CT is a tryptophan auxotroph and cannot synthesize tryptophan de novo, but only via indole salvage [38]. Therefore, increased biosynthesis of chorismate, a precursor of indole, indole derivatives, and tryptophan, could be attributed to the peculiar metabolic needs of *Chlamydia*. Instead, the increased involvement in mixed acid fermentation could reflect a dysbiotic lactobacilli-depleted vaginal ecosystem associated with CT infection. Indeed, BV status is characterized by the presence of many facultative and strict anaerobes, able to produce different volatile and non-volatile organic acids, also as a result of mixed acid sugar fermentation [39].

Future studies should address the vaginal and anal microbiomes composition in more women with and without CT. Studies with a larger cohort of subjects will be able to investigate other possible influences on these ecological niches, including but not limited to ethnicity as well as contraceptive and recent antibiotic use, which were strategically excluded from the current study to reduce variability between women.

The outcome of this study could be useful to set up new diagnostic/prognostic tools, to find correlations with the presence of peculiar clinical or behavioral traits, and to evaluate the possibility of a different susceptibility to chlamydial STIs based on microbiome composition. Thus, intriguing perspectives on the control of chlamydial infections, in terms of prevention and treatment, could be investigated. For example, the study of vaginal and anal microbiome composition could help predict a higher risk of CT infection, implementing strategies for CT prevention in specific subgroups of women.

Moreover, it could be possible to design the use of oral and/or vaginal probiotics (e.g., composed of lactobacilli) to prevent CT acquisition or to help clear the infection, along with antibiotic treatment.

Further investigations with a prospective longitudinal study design would afford understanding whether these alterations precede the infection onset or if the pathogen itself perturbs the vaginal and anal environment.

## 4. Materials and Methods

### 4.1. Study Population and Sample Collection

Eligible subjects were selected from a group of Caucasian non-pregnant young women attending the STI Outpatients Clinic of S. Orsola-Malpighi Hospital in Bologna (Italy) between May and December 2019 and reporting unsafe sexual intercourse. Criteria for the enrollment were as follows: (i) absence of ano-genital symptoms and (ii) presence of risk factors for CT infections (i.e., new, or multiple sexual partners, unprotected intercourse, history of a partner positive for STIs).

Exclusion criteria included: being under the age of 18 years or over 26; having used antibiotic treatments in the month before the study; presence of inflammatory bowel diseases (IBD); presence of infectious intestinal pathologies; or use of enemas within 3 days before sampling. Moreover, samples positive for *Neisseria gonorrhoeae*, *Mycoplasma genitalium*, HSV, and *Treponema pallidum* were excluded from the study. For each patient, personal and demographic data were recorded, and a clinical examination was carried out. Vaginal and an anal swab (E-Swab, Copan, Brescia, Italy) were collected from each woman for the detection of CT and the other STI pathogens and for the profiling of the microbiome. The adequacy of mucosal sampling in terms of cellularity degree was confirmed by means of PCR, targeting the human beta-globin gene [40].

The study protocol was reviewed and approved by the Ethical Committee of St. Orsola-Malpighi Hospital (7/2016/U/Tess). Written informed consent to the work was collected from all subjects.

### 4.2. Diagnosis of Ano-Genital Infections

Vaginal and anal swabs were processed by Versant CT/GC DNA 1.0 Assay (Siemens Healthineers, Tarrytown, NY, USA), a duplex real-time PCR test assessing the presence of CT and *N. gonorrhoeae* DNA, as described in Marangoni et al. [41]. Each sample was tested for *Mycoplasma genitalium* with a quantitative PCR assay [42]. HSV and *T. pallidum* infections were excluded by means of a multiplex molecular approach (FTD genital ulcer, Fast Track Diagnostics, Esch sur Alzette, Luxembourg).

Eligible subjects were allocated in one of the following groups: CT-negative (negativity for both vaginal and anal *Chlamydia*), and CT-positive (positivity both for vaginal and anal samples).

### 4.3. Analysis of the Vaginal and Anal Microbiome

Starting from the remaining DNA eluate of the Versant PCR plate, the V3–V4 hypervariable regions of the bacterial 16S rRNA gene were amplified according to the 16S metagenomic sequencing library preparation protocol (Illumina, San Diego, CA, USA) and sequenced on a MiSeq platform (Illumina) in a single 2 × 300 bp paired-end run.

Raw sequences were analyzed with the QIIME 2.0 pipeline, version 2020.11 [43]. The sequences were trimmed for primer removal with Cutadapt [44] and denoised with DADA2 to obtain ASVs [45]. Taxonomy assignment was carried out with the feature classifier VSEARCH [46], with SILVA SSU database release 138 (https://www.arb-silva.de/download/arb-files/; accessed on 28 September 2021) as reference and the similarity threshold set at 0.97. ASVs identified as “*Lactobacillus*_uncultured” were further analyzed with a BLAST search into NCBI 16S ribosomal RNA sequences database to assign species level. The feature table was rarefied to the lowest number of reads (6412 per sample) to compute and compare, with the appropriate QIIME2 plugins, alpha- (Chao1, Shannon, and Pielou’s evenness) and beta-diversity (Weighted UniFrac) (i.e., Kruskal–Wallis test and PERMANOVA for alpha- and beta diversity, respectively). Differences were considered significant for *p* < 0.05. PCoA was computed with QIIME2, based on the beta-diversity distance matrix.

The taxonomic profiles of vaginal microbial communities were analyzed by VALENCIA, a nearest centroid-based tool that classifies samples according to the similarity to a set of 13 reference CSTs [24]. 

Linear discriminant analysis effect size (LEfSe, http://huttenhower.sph.harvard.edu/galaxy; accessed on 28 September 2021) algorithm was utilized to discover distinctive taxonomic features characterizing CT-positive and negative samples [47]. Taxa presenting a significant differential abundance (*p* < 0.05) and logarithmic LDA (linear discriminant analysis) score > 2 were considered microbial biomarkers of CT-positive or -negative samples.

Correlations between *Lactobacillus* spp., *G. vaginalis*, CT, and other bacterial taxa identified in vaginal samples were determined using sparse co-occurrence network investigation for compositional data (SCNIC) (https://github.com/shafferm/SCNIC; accessed on 28 September 2021) by computing Spearman correlation coefficients (*p* < 0.05) [48].

The vaginal dataset was analyzed with PICRUSt2 to predict metagenome functions and investigate significant differences between CT-positive and -negative samples in terms of metabolic pathways [49]. The tool predicted the enzymes and the relative MetaCyc pathways abundances [50]. STAMP was used to visualize the results and perform statistical analysis [51].

## Data Availability

The 16S rRNA gene sequences datasets are available in the NCBI repository with the BioProject ID: PRJNA764037 (https://www.ncbi.nlm.nih.gov/; accessed on 28 September 2021). Other data presented in this study are available in the Supplementary Materials.

## Supplementary Materials

The following are available online at https://www.mdpi.com/article/10.3390/pathogens10101347/s1, Figure S1: Alpha diversity metrics of anal and vaginal microbiome, Figure S2: Classification of vaginal microbial communities in VALENCIA community state types, Figure S3: Microbial sharing among vaginal and anal microbiota, Figure S4: Taxonomic biomarkers characterizing CT-positive and negative samples. Figure S5: Metabolic functional analysis prediction of anal samples.
