# Supplementary material for: Vaginal and Anal Microbiome during Chlamydia trachomatis Infections
Source: Pathogens. 2021 Oct 19;10(10):1347. doi: 10.3390/pathogens10101347 (PMC8539191; doi:10.3390/pathogens10101347)
Supplement: Supplementary file 1 [file pathogens-10-01347-s001.zip › pathogens-1420095-supplementary.pdf]

Supplementary Materials

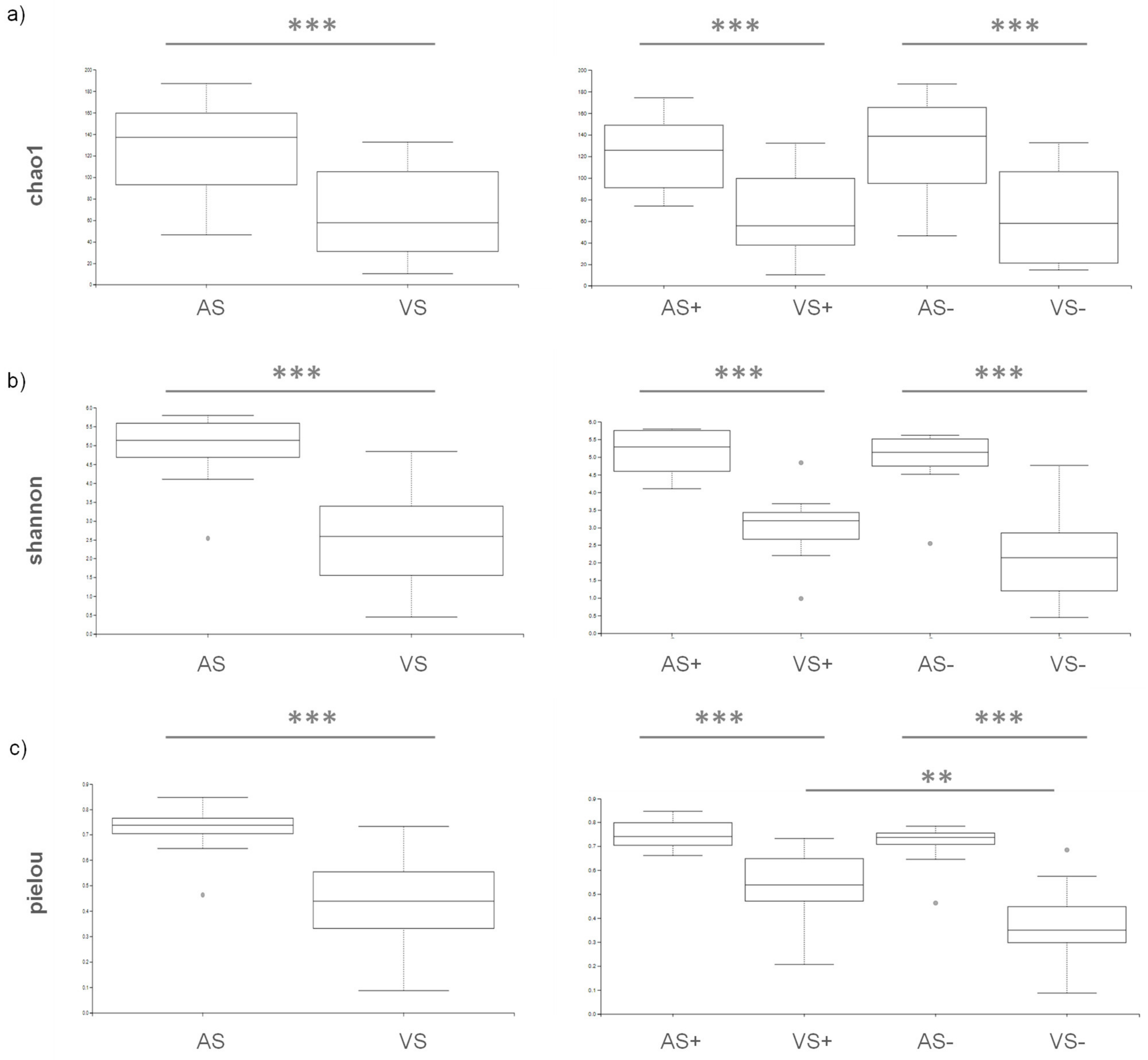

Figure S1. Alpha diversity metrics of anal and vaginal microbiome. Metrics, comprising Chao1, Shannon, and Pielou indexes are calculated and the median, the 25th and 75th percentiles (box), the 10th and 90th percentiles (whiskers) are indicated. On the left, metrics were obtained grouping all anal and vaginal samples, irrespective of chlamydia positivity. On the right values were calculated stratifying samples for CT positivity. Significant differences according to Kruskal-Wallis test for $p < 0.0001$ (***) and $p < 0.01$ (**) are reported.

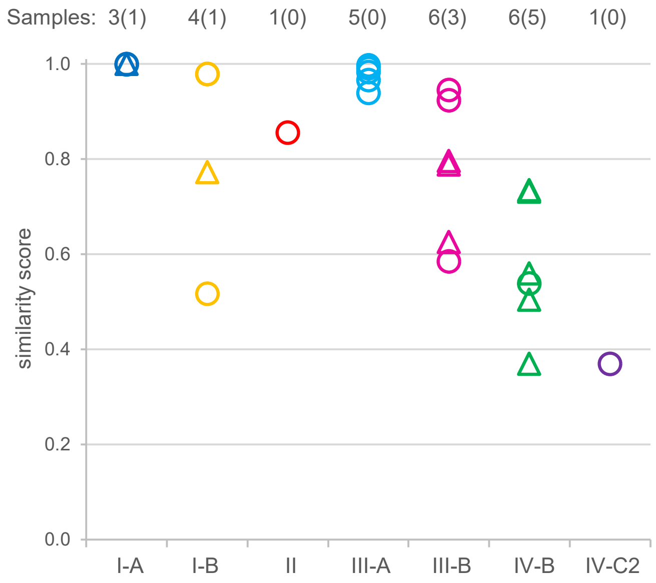

Figure S2. Classification of vaginal microbial communities in VALENCIA community state types. The taxonomic profiles of vaginal microbial communities were analyzed by VALENCIA algorithm, classifying samples according to the similarity to a set of 13 reference community state types (CSTs). The similarity score of each CT-positive (triangles) and CT-negative (circles) sample, calculated with respect to the corresponding CTS, is reported.

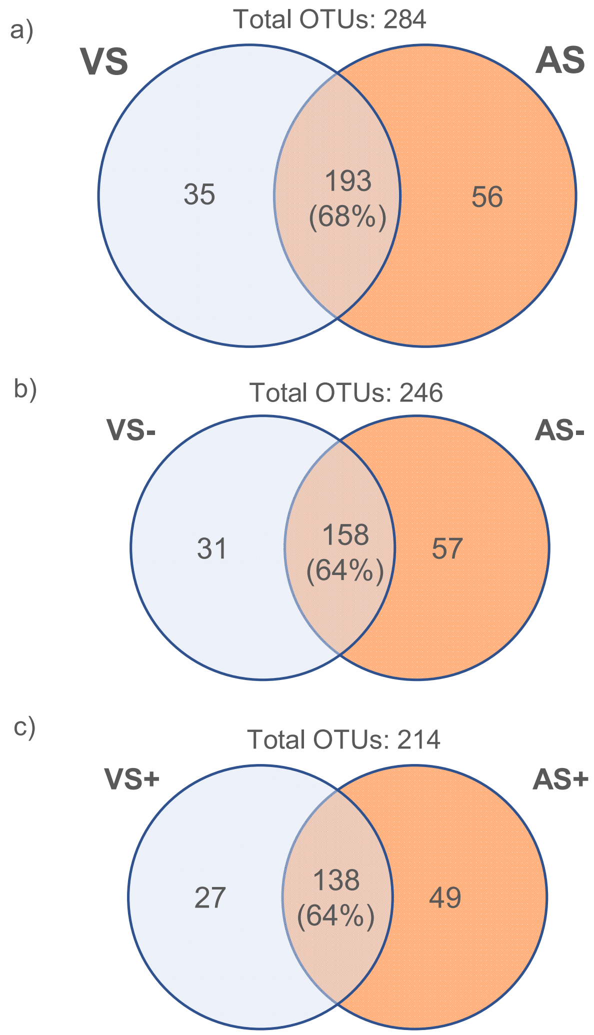

Figure S3. Microbial sharing among vaginal and anal microbiota. Distribution of the identified OTUs among vagina (blue set) and anus (orange set). OTUs shared by at least one sample of each niche were reported in the intersection. Relationship between all anal and vaginal samples (a), CT-negative (b), and CT-positive (c) groups are reported.

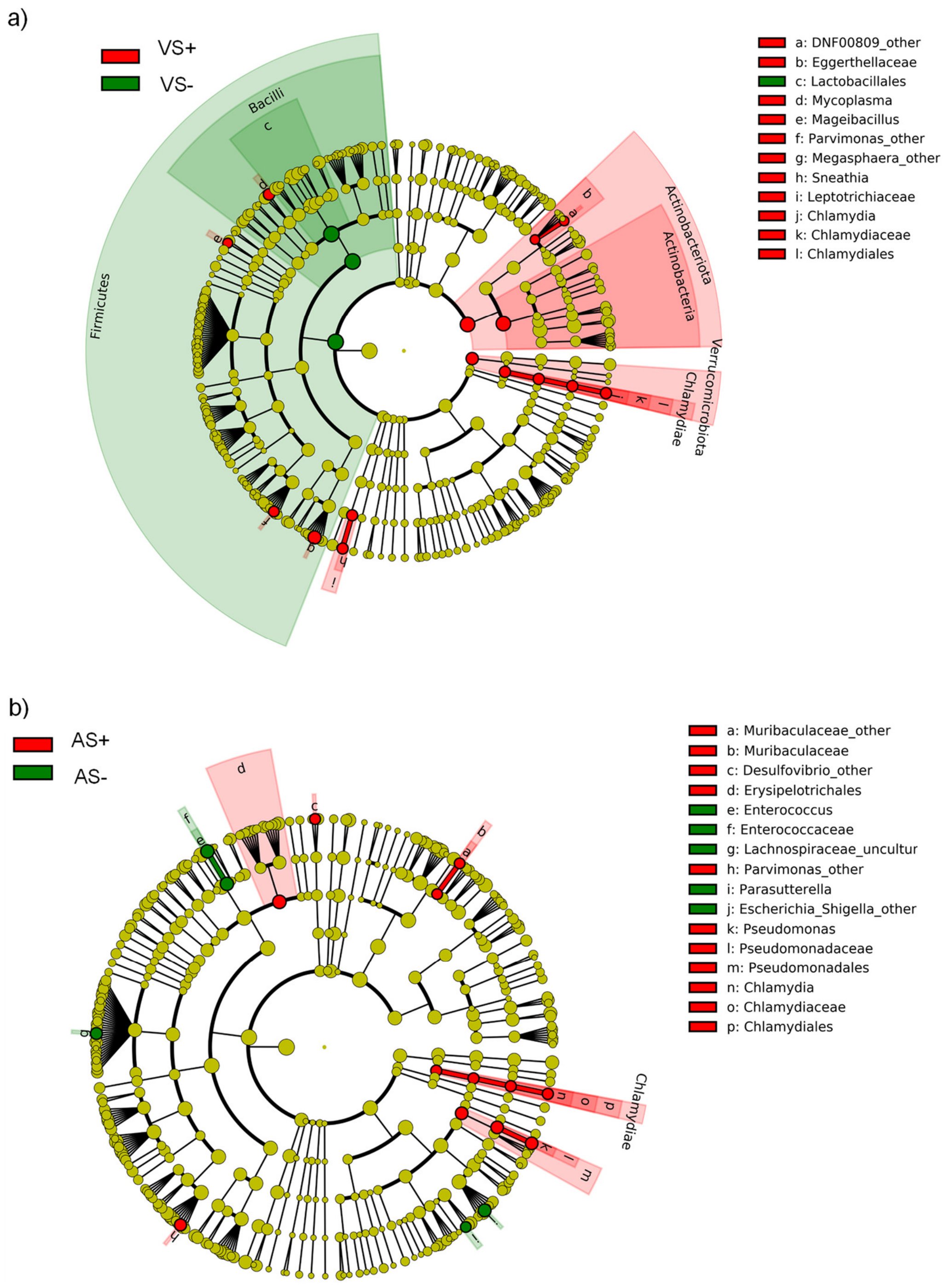

Figure S4. Taxonomic biomarkers characterizing CT-positive and negative samples. Cladogram visualization of the distinctive features characterizing vaginal (a) and anal (b) samples according to LEfSe analysis. Taxa exhibiting significant differential abundance ($p < 0.05$) between CT-positive and CT-negative groups are reported.
